# US Prison Policies on Organ Donation for Individuals Who Are Incarcerated

**DOI:** 10.1001/jamanetworkopen.2023.2047

**Published:** 2023-03-08

**Authors:** Yoshiko Iwai, Michael Forrest Behne, Jason M. Long, Lauren Brinkley-Rubinstein

**Affiliations:** 1 currently a medical student at University of North Carolina School of Medicine at Chapel Hill; 2 Center for Health Equity Research, Department of Social Medicine, University of North Carolina at Chapel Hill; 3 currently a graduate student at University of North Carolina Gillings School of Global Public Health, Chapel Hill; 4 Division of Cardiothoracic Surgery, Department of Surgery, University of North Carolina at Chapel Hill; 5 currently affiliated with Department of Population Health Sciences, Duke University, Durham, North Carolina

## Abstract

This cross-sectional study collects data on US prison policies concerning organ donation by incarcerated individuals.

## Introduction

Organ donation in prisons is ethically fraught. The Organ Procurement and Transplantation Network states, “absent any societal imperative, one’s status as a prisoner should not preclude them from consideration for a transplant.”^1^ Organ donation ethics for incarcerated individuals have been debated, particularly around sentence reduction and discriminatory consequences.^2,3^ Bioethics literature underscores the complexity of supply-demand needs in the face of autonomy, practical barriers, and moral concerns.^4,5^ We collected data on US prison policies surrounding organ donation.

## Methods

Publicly available operational policies were reviewed for 50 state prisons; the jail in Washington, DC; the Federal Bureau of Prisons; and Immigration and Customs Enforcement. Policies were accessed from governmental websites (eTable in Supplement 1) from May 1 to June 30, 2022. A policy analyst (M.F.B.) and a second researcher (L.B.R.) reviewed policies using an inductive coding approach. Date of policy publication and/or review was recorded. Because this study was considered nonhuman participant research, the need for approval and informed consent was waived by the University of North Carolina Institutional Review Board. The study followed the STROBE reporting guideline.

## Results

Organ donation and transplant policies for 53 carceral systems were abstracted (Table). Twenty-one systems had available policies. Fifteen systems permitted individuals to donate while living and 13 permitted registering for posthumous donation. Twelve systems limited donation to family, including the Federal Bureau of Prisons, which specified permissions for living donation to immediate family only. Pennsylvania granted the warden authority to donate. Family notification was codified in Iowa, New York, and Washington. Language used in the eligibility criteria varied (Table).

**Table.** State Policies on Organ Donation for Individuals Who Are Incarcerated

Jurisdiction	Policy	Date of policy effect/revision	Summary	Policy				
				Donate while alive	Register for posthumous donation	Warden authority to donate	Family notification codified	Blood donation (Y/N)
Federal BOP	Yes	June 3, 2014	Living donation to immediate family, not allowed to register for posthumous donation	Yes	No	NR	No	No
ICE	No	NA	NA	NA	NA	NA	NA	NR
Alabama	No	NA	NA	NA	NA	NA	NA	NR
Alaska	Yes	March 3, 1986	Living donation to immediate family, “nothing in this policy precludes a prisoner's making a [posthumous] anatomical gift”	Yes	Yes	NR	No	NR
Arizona	Yes	October 1, 2022	Living donation to immediate family, program to allow and facilitate donation	Yes	Yes	NR	No	No
Arkansas	No	NA	NA	NA	NA	NA	NA	NR
California	Yes	June 2018	Can register for posthumous donation but living gifts not covered	Unclear	Yes	NR	No	Yes
Colorado	No	NA	NA	NA	NA	NA	NA	NR
Connecticut	No	NA	Warden will consult with next of kin for disposition of remains	NA	NA	NA	NA	NR
Delaware	No	NA	NA	NA	NA	NA	NA	NR
Florida	No	NA	NA	NA	NA	NA	NA	NR
Georgia	Yes	February 1, 2022	Living donation to immediate family, does not preclude donation or registration but legal requirements for autopsies may prevent organ procurement	Yes	Unclear	NR	No	NR
Hawaii	No	NA	NA	NA	NA	NA	NA	NR
Idaho	Yes	February 2001	Only in the case of an immediate family	Yes	No	NR	No	NR
Illinois	Yes	March 1, 2005	No	No	NR	NR	NR	NR
Indiana	No	NA	NA	NA	NA	NA	NA	NR
Iowa	Yes	July 2021	Must notify state donor network and family by law	No	Yes	No	Yes	NR
Kansas	No	NA	NA	NA	NA	NA	NA	NR
Kentucky	No	NA	NA	NA	NA	NA	NA	NR
Louisiana	No	NA	NA	NA	NA	NA	NA	NR
Maine	No	NA	NA	NA	NA	NA	NA	NR
Maryland	Yes	February 20, 2016	Living donation to immediate in-state family	Yes	Unclear	NR	NR	Yes
Massachusetts	No	NA	NA	NA	NA	NA	NA	NA
Michigan	Yes	June 28, 2021	Are eligible, scant details	Yes	Yes	NR	NR	NR
Minnesota	Yes	March 20, 2018	Eligible for blood, marrow, or organ donation if “vital in saving another person's life”	Yes	NR	NR	NR	Yes
Mississippi	No	NA	NA	NA	NA	NA	NA	NR
Missouri	No	NA	NA	NA	NA	NA	NA	NR
Montana	No	NA	NA	NA	NA	NA	NA	NR
Nebraska	No	NA	NA	NA	NA	NA	NA	NR
Nevada	Yes	May 15, 2018	Living donation to immediate family if only available option, posthumous donation registration allowed	Yes	Yes	No	NR	No
New Hampshire	No	NA	No	No	No	No	No	NR
New Jersey	No	NA	No	No	No	No	No	NR
New Mexico	No	NA	No	No	No	No	No	NR
New York	Yes	April 29, 2022	As living donors to family or with special approval and in cases of brain death with family's consent	Yes	NR	No	Yes	NR
North Carolina	Yes	January 2009	“May request to be organ donors”	Yes	Yes	No	No	NR
North Dakota	No	NA	NA	NA	NA	NA	NA	NR
Ohio	Yes	September 15, 2016	Living donation to family members (and other by director’s approval), and program for posthumous organ donation	Yes	Yes	No	NR	NR
Oklahoma	No	NA	NA	NA	NA	NA	NA	NR
Oregon	No	NA	Disposition of remains policies gives latitude for individual, family, and DOC to initiate donation procedures	NA	NA	NA	NA	NR
Pennsylvania	Yes	January 12, 2022	Donor registration available, in absence of this the DOC may be able to intercede	No	Yes	Yes	No	NR
Puerto Rico	No	NA	NA	NA	NA	NA	NA	NR
Rhode Island	No	NA	Not in publicly available policies	NA	NA	NA	NA	NR
South Carolina	Yes	September 1, 2008	Posthumous donor registration supported	No	Yes	No	No	NR
South Dakota	No	NA	NA	NA	NA	NA	NA	NR
Tennessee	No	NA	NA	NA	NA	NA	NA	NR
Texas	Yes	April 2022	Posthumous donor registration supported	No	Yes	No	No	NR
Utah	No	NA	NA	NA	NA	NA	NA	NR
Vermont	No	NA	NA	NA	NA	NA	NA	NR
Virginia	Yes	October 1, 2019	Living donation to family in absence of other donors	Yes	NR	No	No	NR
Washington	Yes	July 29, 2021	Living donation of blood and organs to family, posthumous donation supported	Yes	Yes	No	Yes	Yes
Washington, DC	No	NA	NA	NA	NA	NA	NA	NR
West Virginia	No	NA	NA	NA	NA	NA	NA	NR
Wisconsin	Yes	August 8, 2014	Living donation to relatives, posthumous donation permitted	Yes	Yes	No	No	NR
Wyoming	No	NA	NA	NA	NA	NA	NA	NR

Abbreviations: BOP, Bureau of Prisons; DOC, Department of Corrections; ICE, Immigration and Customs Enforcement; NA, not applicable; NR, not reported.

Of the 27 states with capital punishment, 10 maintained donation-related policies. Arizona, Nevada, and Ohio made explicit references to organ and tissue donation after completion of an execution warrant; Arizona and Nevada excluded donation and procurement from individuals facing execution, while Ohio abdicated responsibility for such decisions.

The oldest policy (Alaska’s) was enacted March 3, 1986, and the newest policy (Arizona’s) was reviewed October 1, 2022. Sixteen systems (80%) had reviewed policies within the past 10 years, and 8 policies were operationalized within 2 years.

## Discussion

Only 40% of US carceral systems had available policies on organ donation, and it was often unclear which authority gave final donation permissions. Additionally, ethical implications around organ and tissue donation following executions are not well-defined in Department of Corrections (DOC) policy. Although many policies were outdated, nearly half of the systems with policy review in the last 10 years took place since 2020. This suggests growing concern among DOCs, perhaps owing to a rapidly aging confined population and COVID-19.

Assessing the current state of organ donation in prisons is challenging. Hospitals managed by carceral agencies are not uniformly obligated to share data with federal organizations, and most do not self-report outcomes. We had intended to analyze donor and transplant rates in conjunction with policies; however, procurement networks do not collect incarceration status. Standardized transplant policies could include data reporting provisions to aid researchers in addressing health inequities.

In early 2023, state legislators in Massachusetts introduced a bill that would allow people who are incarcerated to donate their organs in exchange for the prospect of early release.^6^ It is essential that organizations like the United Network for Organ Sharing develop clear guidelines that ensure ethical practices that do not result in exploitation, and advocate for community carceral parity. Uncertainty faced by transplant stakeholders may be mitigated by clearer guidelines from DOCs and the Organ Procurement and Transplantation Network. In many cases, these policies are not accessible; thus, serious consideration should be given when transplants involve individuals with incarceration history.

Limitations of this study include its lack of generalizability to other carceral systems and countries. We also acknowledge that some organ donation policies may not have been available during our data collection period and were therefore omitted from our analysis. Additionally, we could not discern transplant need and rates in prisons owing to lack of transplant data.

The findings of this cross-sectional study suggest that US prisons vary in organ donation policies, with only 40% of systems having accessible policies. Improved data collection and reporting mechanisms are critical for ensuring equity.

## Notes

Supplement 1. **eTable.** List of Websites for Data Collection

Supplement 2.
Data Sharing Statement
