# Supplementary material for: US Prison Policies on Organ Donation for Individuals Who Are Incarcerated
Source: JAMA Netw Open. 2023 Mar 8;6(3):e232047. doi: 10.1001/jamanetworkopen.2023.2047 (PMC9996393; doi:10.1001/jamanetworkopen.2023.2047)
Supplement: Supplement 1. — eTable. List of Websites for Data Collection [file jamanetwopen-e232047-s001.pdf]

Supplementary Online Content

Iwai Y, Behne MF, Long JM, Brinkley-Rubinstein L. US prison policies on organ donation for individuals who are incarcerated. *JAMA Netw Open*. 2023;6(3):e232047. doi:10.1001/jamanetworkopen.2023.2047

eTable. List of Websites for Data Collection

This supplementary material has been provided by the authors to give readers additional information about their work.

© 2023 Iwai Y et al. *JAMA Network Open*.

eTable. List of Websites for Data Collection

Jurisdiction	Website Available (Y/N)	Website
**Federal BOP**	Y	https://www.bop.gov/policy/progstat/6031_004.pdf
**ICE**	N	
**Alabama**	N	
**Alaska**	Y	https://doc.alaska.gov/pnp/pdf/807.10.pdf?TSPD_101_R0=0890181cafab200086badf7858c2ded260d224fe9cdc59a1fd49ab81a913694583d4976985d81da308c2b5e3be14300094c41bbc5a61db8ace61bc0511b5068b19c0d4db6ad14f8369946bcfe5a4bbd0150314a5c3504e341ce057cf14d55d8c
**Arizona**	Y	https://corrections.az.gov/sites/default/files/documents/policies/900/0922.pdf
**Arkansas**	N	
**California**	Y	https://cchcs.ca.gov/wp-content/uploads/sites/60/HC/HCDOM-ch02-art4.1.pdf
**Colorado**	N	
**Connecticut**	N	
**Delaware**	N	
**Florida**	N	
**Georgia**	Y	https://public.powerdms.com/GADOC/documents/106402
**Hawaii**	N	
**Idaho**	Y	http://forms.idoc.idaho.gov/WebLink/ElectronicFile.aspx?docid=273637&dbid=0
**Illinois**	Y	https://www.ilga.gov/commission/jcar/admincode/020/020004150000800R.html
**Indiana**	N	
**Iowa**	Y	https://doc.iowa.gov/sites/default/files/fph-21_organ_procurement_1.pdf
**Kansas**	N	
**Kentucky**	N	
**Louisiana**	N	
**Maine**	N	
**Maryland**	Y	https://itcd.dpscs.state.md.us/PIA/ShowFile.aspx?fileID=697
**Massachusetts**	N	
**Michigan**	Y	https://www.michigan.gov/corrections/-/media/Project/Websites/corrections/Files/Policy-Directives/PDs-04-Institutional-Operations/PD-0406-Medical-and-Mental-Health-Services/04-06-110-Deaths-and-Disposition-of-the-Remains-effective-06-28-21.pdf?rev=190cc685e9b546d1b9fb4591557f8965
**Minnesota**	Y	https://policy.doc.mn.gov/DOCPolicy/
**Mississippi**	N	
**Missouri**	N	
**Montana**	N	
**Nebraska**	N	

© 2023 Iwai Y et al. *JAMA Network Open*.

**Nevada**	Y	https://doc.nv.gov/uploadedFiles/docnvgov/content/About/Administrative_Regulations/AR 659 Inmate Organ Donor - Final - 05152018.pdf
**New Hampshire**	N	
**New Jersey**	N	
**New Mexico**	N	
**New York**	Y	https://doccs.ny.gov/system/files/documents/2022/05/4330.pdf
**North Carolina**	Y	https://files.nc.gov/ncdps/documents/files/adv3.pdf
**North Dakota**	N	
**Ohio**	Y	https://drc.ohio.gov/Portals/0/Policies/DRC Policies/68-MED-09 (Sept 2016).pdf?ver=2016-10-12-141116-720
**Oklahoma**	N	
**Oregon**	N	
**Pennsylvania**	Y	https://www.cor.pa.gov/About Us/Documents/DOC Policies/13.01.01 Management and Administration of Health Care.pdf
**Puerto Rico**	N	
**Rhode Island**	N	
**South Carolina**	Y	https://www.doc.sc.gov/policy/HS-18-01.htm.pdf
**South Dakota**	N	
**Tennessee**	N	
**Texas**	Y	https://www.tdcj.texas.gov/divisions/cmhc/docs/cmhc_policy_manual/E-31.02.pdf
**Utah**	N	
**Vermont**	N	
**Virginia**	Y	https://vadoc.virginia.gov/files/operating-procedures/700/vadoc-op-750-2.pdf
**Washington**	Y	https://www.doc.wa.gov/information/policies/default.aspx?show=600
**Washington, D.C.**	N	
**West Virginia**	N	
**Wisconsin**	Y	https://doc.wi.gov/DepartmentPoliciesDAI/5003013.pdf
**Wyoming**	N	

© 2023 Iwai Y et al. *JAMA Network Open*.
