# Supplementary material for: US Prison Policies on Organ Donation for Individuals Who Are Incarcerated
Source: JAMA Netw Open. 2023 Mar 8;6(3):e232047. doi: 10.1001/jamanetworkopen.2023.2047 (PMC9996393; doi:10.1001/jamanetworkopen.2023.2047)
Supplement: Supplement 2. — Data Sharing Statement [file jamanetwopen-e232047-s002.pdf]

Data Sharing Statement

Iwai. US Prison Policies on Organ Donation for Individuals Who Are Incarcerated. *JAMA Netw Open*. Published March 08, 2023. doi:10.1001/jamanetworkopen.2023.2047

Data
Data available: No
